# Do-not-attempt-resuscitation orders: attitudes, perceptions and practices of Swedish physicians and nurses

**DOI:** 10.1186/s12910-021-00604-8

**Published:** 2021-03-30

**Authors:** Anders Bremer, Kristofer Årestedt, Ewa Rosengren, Jörg Carlsson, Samuel Sandboge

**Affiliations:** 1 Department of Health and Caring Sciences, Faculty of Health and Life Sciences, Linnaeus University, Universitetsplatsen 1, 35195 Växjö, Sweden; 2 Department of Ambulance Service, Region Kalmar County, Lasarettsvägen 37, 39244 Kalmar, Sweden; 3 University of Borås, Allégatan 1, 50190 Borås, Sweden; 4 The Research Section, Region Kalmar County, Box 601, 39126 Kalmar, Sweden; 5 The Cardio Thoracic Intensive Care Unit, Karolinska University Hospital, Region Stockholm, Eugeniavägen 23, 17164 Solna, Sweden; 6 Department of Medicine, Section of Cardiology, Kalmar County Hospital, Box 601, 39126 Kalmar, Sweden; 7 Population Health Unit, Finnish Institute for Health and Welfare, Box 30, 00271 Helsinki, Finland

**Keywords:** Attitudes, Autonomy, Cardiac arrest, DNAR order, Informed consent, Nurses, Physicians

## Abstract

**Background:**

The values and attitudes of healthcare professionals influence their handling of ‘do-not-attempt-resuscitation’ (DNAR) orders. The aim of this study was a) to describe attitudes, perceptions and practices among Swedish physicians and nurses towards discussing cardiopulmonary resuscitation and DNAR orders with patients and their relatives, and b) to investigate if the physicians and nurses were familiar with the national ethical guidelines for cardiopulmonary resuscitation.

**Methods:**

This was a retrospective observational study based on a questionnaire and was conducted at 19 wards in two regional hospitals and one county hospital.

**Results:**

210 physicians and 312 nurses (n = 522) responded to the questionnaire. Every third (35%) professional had read the guidelines with a lower proportion of physicians (29%) compared to nurses (38%). Around 40% of patients had the opportunity or ability to participate in the DNAR discussion. The DNAR decision was discussed with 38% of patients and the prognosis with 46%. Of the patients who were considered to have the ability to participate in the discussion, 79% did so. The majority (81%) of physicians and nurses believed that patients should always be asked about their preferences before a DNAR decision was made.

**Conclusions:**

Swedish healthcare professionals take a patient’s autonomy into account regarding DNAR decisions. Nevertheless, as 50% of patients were considered unable to participate in the DNAR discussion, questions remain about the timing of patient participation and whether more discussions could have been conducted earlier. Given the uncertainty about timing, the majority of patients deemed competent participated in DNAR discussions.

**Supplementary Information:**

The online version contains supplementary material available at 10.1186/s12910-021-00604-8.

## Introduction

Medical and technical advances have made it possible to prolong patients’ lives, even in the case of fatal diseases and serious conditions. However, valuable medical knowledge and technology can be harmful if they are used for life-saving purposes when treatment only prolongs a patient’s suffering and compromises their dignity [1]. Thus, end-of-life care planning is essential in order to avoid medical treatment that does not benefit the patient and to respect the patient’s right to refuse life-prolonging treatment [2]. Such planning enables care based on end-of-life conversations that take into account and respects the patient’s dignity, including their preferences, wishes, values and beliefs [3, 4]. Important aspects of this planning include the provision of care in accordance with the principles of beneficence and non-maleficence with the intention of acting in the best interests of the patient [5].

Structured advance care planning programmes have been shown to improve the congruence between a patient and their family regarding end-of-life care preferences. The planning also reduces the patient’s decisional conflict and increases the documentation of care preferences [6]. End-of-life decisions are increasingly seen as a form of teamwork in which physicians and nurses work together with patients and family members to reach a decision that reflects the preferences and values of the patient, while also taking into account the medical aspects [7]. As part of strengthening the patient’s autonomy in the end-of-life care planning, the issue of CPR and the patient’s preferences for ‘do-not-attempt-resuscitation’ (DNAR) orders is supported in most European countries [8]. However, there is a difference between how end-of-life care planning, including discussions about DNAR, should work and how it works in reality.

In a systematic review [9], senior physicians were identified as key decision makers and responsible for making DNAR decisions, while nurses played a key role in initiating and following up discussions about DNAR. At the same time, a number of barriers to initiating discussions with patients were identified. For example, physicians felt discomfort, embarrassment, unskilled, inadequately trained and/or inexperienced. They also reported low level of confidence, concerns about complaints and difficulty in making a decision. The barriers for nurses to discussing DNAR were concerns about harming the patient and lacking courage to withdraw treatment due to medical uncertainty [9].

With the aim of promoting the patient’s participation in conversations about DNAR and protecting patient autonomy and fair treatment, the European Resuscitation Council has formulated guidelines that promote the protection of a patient’s preferences and to make decisions that are in accordance with the patient’s values and beliefs [8]. Based on these guidelines, it is often necessary to formulate national and local guidelines that can be applied to a clinical setting [10]. In 2011, Swedish healthcare professionals also indicated that there were differences in the way decisions about cardiopulmonary resuscitation (CPR) were made and documented, which led to requests for new national ethical guidelines [11]. Thus, two years later, the Swedish Society of Medicine, the Swedish Society of Nursing, and the Swedish Resuscitation Council published national ethical guidelines for CPR [12]. Focusing on patient autonomy, these guidelines state that the wishes of a patient, who is competent to make decisions, properly informed, and who understands the consequences of refusing CPR, should be respected [12]. However, in practice, ensuring that patient autonomy is respected, while also being confident that the decision is in the patient’s best interests, can be complex and challenging [13]. Also, the values of healthcare professionals can vary. Further, patient involvement in discussing DNAR varies between countries and care contexts and is reported to be between 25 and 82% [13–16]. In Sweden in the late 1990s, around one third of patients deemed competent to make a decision regarding a DNAR order participated in the discussion [17]. Fifteen years later, discussions were still at the same level [18] and around one half of professionals reported that it was unlikely that patients would be involved in DNAR decisions [19].

To summarise, thus far the focus has been on studying barriers and facilitators to discussions with patients about DNAR from separate professional perspectives, which makes it difficult to compare similarities and differences between the key decision makers. The aim of this study was therefore to describe attitudes, perceptions and practices among Swedish physicians and nurses towards discussing DNAR orders with patients and their relatives, and to investigate if the physicians and nurses were familiar with the national ethical guidelines.

## Methods

This study conforms to the ethical principles for medical research involving human subjects outlined in the Declaration of Helsinki [20] and adheres to Swedish laws and regulations concerning research, informed consent, and confidentiality [21]. This retrospective observational study is based on an online questionnaire. The study was approved by the Regional Ethical Review Board in Gothenburg in Sweden (No. 636-17), also including the research plan, the information to the participants, and the questionnaire. The heads of operations in all participating departments approved that the study was conducted, and that the invitation letter was sent to the physicians and nurses employed in the departments. Informed consent was obtained from all subjects involved in this study.

### Settings and study population

Patient autonomy plays a key role in Swedish healthcare legislation [22, 23]. When a patient who has a decision-making ability, has assimilated the available information and understands the consequences of the various treatment options, states that they do not wish life-sustaining treatment to be initiated or terminated, healthcare professionals must respect this wish. This also applies to situations in which the patient is not in the final stages of life and in which treatment could medically benefit them. Relatives have a subordinate role. When a patient is incapable of making decisions, and there are no oral or written directives documenting the patient’s wishes, healthcare professionals should consult with those who know the patient best in order to try and establish what the patient would have wanted if they had been able to make decisions themselves. It is usually the patient’s relatives who are consulted. The senior physician has ultimate responsibility for the decision to either refrain from administering or terminate life-sustaining treatment [24].

Data collection was conducted between October 2017 and February 2018. Hospitals from three different regions were included to achieve a varied selection of participants across specialities and departments. An online questionnaire was sent via email to 888 physicians and 2189 nurses at 19 departments in two regional hospitals and one county hospital comprising a total of around 3400 beds. The departments comprised internal medicine and related specialities (e.g. rheumatology and infectious diseases), psychiatry, surgical specialities, anaesthesiology and intensive care, as well as emergency medicine.

Email addresses were obtained from the participating departments and/or the hospitals’ administration. An invitation was sent to all potential participants with information about voluntary participation and that completion of the questionnaire implied consent to participate. The participants who agreed to participate were able to access the online questionnaire which was available over a two-month period. A reminder was sent to all participants after one month.

The response rate was 17% (n = 522), 24% (n = 210) among physicians and 14% (n = 312) among nurses. The drop out analysis showed that nurses were less prone to participate than physicians (χ(1) = 39.6, *p* < 0.001).

### Study questionnaire

In order to assess changes in attitudes towards DNAR decisions among healthcare professionals in Sweden over time, the design of the study-specific questionnaire was largely based on a previous design [15, 17, 25]. For the present study, the questionnaire was slightly modified in collaboration with the questionnaire designer, in order to incorporate key aspects of the Swedish national ethical guidelines [12]. Three questions were added relating to the guidelines, resulting in a total of 34 questions after a pilot test (n = 20) to ensure the validity of the questionnaire. The final survey comprised four sections: demographical data; knowledge and practices of DNAR and information to the patient; perceptions and practices of DNAR and information to relatives; and attitudes to DNAR orders in the event of sudden cardiac arrest. The demographic questions were about age, sex, profession, extent of work experience, and type of care specialty. The remaining 28 questions were about DNAR. For the complete questionnaire, see Additional file 1: Attitudes, perceptions and practices to ‘do-not-attempt-resuscitation’ (DNAR) orders.

### Data analysis

Descriptive statistics were used to present sample characteristics, attitudes, perceptions and practices in regard to DNAR, as well as familiarity with the ethical guidelines. Independent sample t-test was used to compare differences in age, years in the profession, and years in health care between physicians and nurses. The Pearson chi-square test was used for all other comparisons between the two groups.

The level of statistical significance was set at *p* < 0.05. All statistical analyses were conducted using Stata 16.1 (StataCorp LLC, College Station, TX, USA).

## Results

The participants’ characteristics and their knowledge and practices regarding the Swedish national ethical guidelines for CPR are presented in Table 1. The mean age in the final sample was 42.0 (SD = 11.5) years. A slightly higher proportion of physicians were men (53%) whereas most nurses were women (85%). The nurses had significantly longer professional experience than the physicians (12.5 vs. 9.2 years, *p* < 0.001).

**Table 1:** Background characteristics and general knowledge and practices regarding DNAR decisions among physicians and nurses

Variables/Questions	All (n = 522)	Physicians (n = 210)	Nurses (n = 312)	*p* value
Age, mean (SD)	42.0 (11.5)	41.9 (11.0)	42.1 (11.9)	0.837 ^a^
Sex, n (%)				< 0.001 ^b^
Female	358 (69.3)	96 (46.2)	262 (84.8)	
Male	156 (30.2)	111 (53.4)	45 (14.6)	
Other	3 (0.6)	1 (0.5)	2 (0.7)	
Years in profession, mean (SD)	11.2 (10.3)	9.2 (8.6)	12.5 (11.1)	< 0.001 ^a^
Years in health care, mean (SD)	16.6 (12.2)	15.0 (10.6)	17.7 (13.1)	0.013 ^a^
Care settings, n (%)				0.001 ^b^
Medicine	243 (46.6)	97 (46.2)	146 (46.8)	
Surgery	39 (7.5)	22 (10.5)	17 (5.5)	
Infection	18 (3.5)	13 (6.2)	5 (1.6)	
Psychiatry	41 (7.9)	12 (5.7)	29 (9.3)	
Emergency	31 (5.9)	9 (4.3)	22 (7.1)	
ICU/Anaesthesia	111 (21.3)	35 (16.7)	76 (24.4)	
Other	39 (7.5)	22 (10.5)	17 (5.5)	
Knowledge of national ethical guidelines, n (%)				0.122 ^b^
Yes	329 (63.0)	124 (59.1)	205 (65.7)	
No	193 (37.0)	86 (41.0)	107 (34.3)	
Have read the guidelines, n (%)				< 0.001 ^b^
Yes	179 (34.6)	61 (29.2)	118 (38.2)	
No	327 (45.8)	119 (56.9)	118 (38.2)	
Uncertain	102 (19.7)	29 (13.9)	73 (23.6)	
Have discussed the guidelines at the clinic, n (%)				0.134 ^b^
Yes	135 (26.0)	46 (22.0)	89 (28.6)	
No	179 (34.4)	81 (38.8)	98 (31.5)	
Uncertain	206 (39.6)	82 (39.2)	124 (39.9)	
Have participated in a discussion leading to a DNAR, n (%)				< 0.001 ^b^
Yes	440 (84.6)	206 (98.6)	234 (75.2)	
No	80 (15.4)	3 (1.4)	77 (24.8)	
Have made a DNAR decision, n (%)				< 0.001 ^b^
Yes	218 (41.8)	183 (87.1)	35 (11.3)	
No	303 (58.2)	27 (12.9)	276 (88.8)	

ICU, Intensive Care Unit; DNAR, Do Not Attempt Resuscitation

^a^Independent sample t-test

^b^Chi-square test

35% of participants had read the national guidelines whereas 63% had heard of them. A significantly lower proportion of physicians had read the guidelines compared to nurses (29% vs. 38%, *p* < 0.001). 99% of physicians and 75% of nurses had participated in a discussion leading to a DNAR decision (*p* < 0.001). 87% of physicians had made such decisions while 11% of nurses stated that they had made a DNAR decision (*p* < 0.001).

### Practices, perceptions and attitudes to DNAR

The practices, perceptions and attitudes toward the participants’ most recent DNAR decision are presented in Table 2. When asked to recall their most recent discussion about a specific DNAR decision, 40% of patients were given the opportunity to participate in the discussion. The DNAR decision was discussed with 38% and the prognosis with 46% of the patients. 79% of patients who were considered able to participate in the discussion, did so (not presented in Table 2). The nurses stated significantly less than physicians that decisions and prognoses were discussed with patients (*p* = 0.042 and *p* = 0.008) and that patients’ opinions were sought (*p* = 0.010).

**Table 2:** Practices, perceptions and attitudes to the most recent DNAR decision: discussion with and information to patient and relatives

Questions	All % (n = 522)			Physicians % (n = 210)			Nurses % (n = 312)			*p* value^a^
	Yes	No	Uncertain	Yes	No	Uncertain	Yes	No	Uncertain	
Was the decision discussed with the patient?	38.4	55.1	6.5	40.8	55.8	3.4	36.4	54.4	9.2	0.042
Was the prognosis discussed with the patient?	45.5	47.5	7.0	50.7	45.8	3.4	40.9	49.0	10.1	0.008
Was the patient asked about his or her opinion in the process that led to the DNAR decision?	35.8	55.2	9.1	40.0	55.1	4.9	32.1	55.3	12.7	0.010
Did the patient have the opportunity or ability to participate in the discussion?	40.1	50.8	9.1	42.2	52.0	5.9	38.4	49.8	11.8	0.093
Was it the patient him- or herself who initiated the discussion?	6.1	87.7	6.1	4.9	93.1	2.0	7.2	83.1	9.7	0.002
Was it the patient him- or herself who requested the DNAR order?	15.9	72.3	11.8	13.8	77.8	8.4	17.7	67.5	14.8	0.040
If the decision was made without the patient’s involvement, was the patient informed of the decision once it was made?	28.2	54.4	17.4	32.2	60.9	6.9	25.0	49.1	25.4	< 0.001
Was the decision ethically right?	90.4	2.3	7.3	93.1	0.5	6.4	88.0	3.9	8.2	0.045
Should the decision have been made earlier?	40.7	34.9	24.4	39.0	39.0	22.0	42.3	31.2	26.5	0.208
Should the decision have been made later?	2.1	82.0	15.9	1.0	87.7	11.3	3.0	77.1	19.9	0.013
Was the decision discussed with the patient’s relatives?	66.9	24.5	8.6	67.5	27.7	4.9	66.4	21.7	11.9	0.019
Was the prognosis discussed with the patient’s relatives?	77.3	15.2	7.5	78.2	16.5	5.3	76.6	14.0	9.4	0.241
Were the patient’s relatives given an opportunity to participate in the discussion?	58.3	27.1	14.6	63.2	29.4	74	54.0	25.1	20.9	< 0.001
Was it the patient’s relatives who initiated the discussion?	4.1	85.4	10.5	3.9	92.2	3.9	4.3	79.5	16.2	< 0.001

DNAR, do not attempt resuscitation

^a^Chi-square test

51% of patients were considered unable to participate in the discussion. When a decision was made *without* patient involvement, physicians stated more often than nurses that patients *had* been informed about the decision (32% vs. 25%, *p* < 0.001). The participants were asked to state why they had not informed the patients. Most participants stated that the patient was unable to participate in a DNAR discussion (54%), either because of critical illness, end-stage dementia, or unconsciousness (not presented in Table 2).

It was rare (6%) for patients to initiate the discussion of a DNAR decision themselves, or to request such a decision to be made (16%). 41% of participants felt that the decision should have been made earlier and 90% considered the decision to have been ethically correct.

The DNAR decision (67%) and the patient’s prognosis (77%) were discussed with relatives while their opinions were considered to a lesser degree (58%) compared to the patient’s opinion. However, a significantly higher proportion of physicians than nurses stated that the relatives were given an opportunity to participate in the discussion (63% vs. 54%, *p* < 0.001).

### Perceptions and attitudes to DNAR

The attitudes about involving patients and/or relatives in DNAR decisions are presented in Table 3. 81% considered that the patient’s opinion should always be sought before making a DNAR decision, whereas 31% stated that a patient’s desire to receive CPR in the event of cardiac arrest should always be respected, regardless of other medical and ethical assessments. A significantly lower proportion of physicians than nurses stated that such a desire should always be respected (16% vs. 41%, *p* < 0.001).

**Table 3:** Perceptions and attitudes of physicians and nurses about involving patients and/or relatives in a DNAR decision

Questions	All (n = 522)	Physicians (n = 210)	Nurses (n = 312)	*p* value ^a^
Should the patient’s opinion about DNAR always be requested on condition that the patient is capable of making a decision?				0.064
Yes	417 (80.8)	157 (75.9)	260 (84.1)	
No	44 (8.5)	22 (10.6)	22 (7.1)	
Uncertain	55 (10.6)	28 (13.5)	27 (8.7)	
Consider a situation in which the patient has expressed a strong desire to receive CPR in the event of sudden cardiac arrest. Should this desire always be respected?				< 0.001
Yes	161 (31.3)	34 (16.4)	127 (41.2)	
No	255 (49.5)	139 (67.2)	116 (37.7)	
Uncertain	99 (19.2)	34 (16.4)	65 (21.1)	
Should the opinions of relatives about DNAR always be requested?				0.588
Yes	319 (61.7)	124 (59.3)	195 (63.3)	
No	97 (18.8)	40 (19.1)	57 (18.5)	
Uncertain	101 (19.5)	45 (21.5)	56 (18.2)	
Should relatives be allowed to make DNAR decisions?				< 0.001
Yes	74 (14.7)	17 (8.4)	57 (19.0)	
No	350 (69.6)	169 (83.3)	181 (60.3)	
Uncertain	79 (15.7)	17 (8.4)	62 (20.7)	
Do you think there are patients who want to be informed that a DNAR decision has been made by the physician in charge but who do not receive such information?				0.617
Yes	422 (81.6)	173 (82.4)	249 (81.1)	
No	14 (2.7)	7 (3.3)	7 (2.3)	
Uncertain	81 (15.7)	30 (14.3)	51 (16.6)	
Do you think there are patients who are informed that a DNAR decision has been made by the physician in charge but who do not want such information?				0.002
Yes	333 (65.0)	152 (74.2)	181 (59.0)	
No	46 (9.0)	15 (7.3)	31 (10.1)	
Uncertain	133 (26.0)	38 (18.5)	95 (30.9)	

CPR, cardiopulmonary resuscitation; DNAR, do not attempt resuscitation

^a^Chi-square test

62% of participants stated that the relatives’ opinions about DNAR decisions should always be requested. A further 15% stated that relatives should be able to make DNAR decisions on behalf of the patient, with significantly less physicians than nurses who believed that relatives should be able to make such a decision (8% vs. 19%, *p* < 0.001).

82% believed that there were occasions when patients who requested information about a DNAR decision did not receive such information. A significant difference was found regarding the opposite, i.e., occasions when patients received information even though they had not requested it, with a higher proportion of physicians than nurses stating that patients were given information they did not want to have (74% vs. 59%, *p* = 0.002).

## Discussion

To the best of our knowledge, this is the largest study to date that uses the same questionnaire to describe attitudes, perceptions and practices among both physicians and nurses regarding discussing and informing patients and relatives about DNAR decisions. The results show that only one third of participants had read the national ethical guidelines for CPR and that physicians had read the guidelines to a significantly lower extent than nurses. Four in five physicians and nurses considered that the patient’s opinion should be heard before making a DNAR decision. Physicians stated to a significantly greater extent than nurses that relatives were heard in the DNAR process and that relatives should not make DNAR decisions. Two in five patients had the opportunity or ability to participate in a DNAR discussion. Of those patients considered to have the ability to participate, four out of five of them of them did so.

### DNAR orders and the impact of the ethical guidelines

In comparison with previous findings [17], Swedish healthcare professionals now appear to be more willing to include patients in DNAR decisions compared to two decades ago. It is unclear whether this has led to an overall increase in the number of DNAR orders in Sweden. However, a recent study [26] suggests that such an increase has taken place as 88.6% of all deaths over a period of one year at a Swedish county hospital had a DNAR order in place.

The low proportion of participants who had read the Swedish ethical guidelines for CPR four years after their publication raises questions about their overall impact. Similar problems in Ireland, where an even lower proportion of healthcare professionals have read the guidelines, have been reported by Brien et al. [27] who found that only 12% of participants had read the Irish National Consent Policy (NCP), which provides a framework for discussing DNAR orders. Interestingly, 11% of the nurses in our study stated that they had made a DNAR decision despite the fact that in Sweden it is usually senior physicians who make formal DNAR decisions, whereas nurses have no legal right to make DNAR decisions. Swedish regulations stipulate that the reason for a DNAR order must be documented and physicians are expected to consult with another healthcare professional before formalising and writing the DNAR order in the patient’s medical record. In practice, this means that the senior physician should preferably consult a colleague or a registered nurse prior to making a decision [24]. This may explain why some nurses felt that they had been making these decisions.

### Practices, perceptions and attitudes regarding DNAR discussions

The present findings indicate that 40% of patients had the opportunity or ability to participate in DNAR discussions. 79% of patients who were considered to have the ability to participate in the discussion did so. The corresponding percentages in a previous Swedish study [17] in a cardiology setting were lower than 18% of patients having the opportunity or ability to participate, while 33% of patients considered to be able to participate did so. Thus, it would appear that Swedish healthcare professionals are more likely to discuss DNAR decisions with patients today compared to two decades ago. If this is the case, it may indicate an increased awareness and ability among professionals to consider the patient’s preferences and autonomy. However, some caution should be exercised when comparing the two studies as our study was conducted in varying care settings.

50% of patients in the present study were considered unable to participate in the discussion, which is a high proportion, given the range of care specialties included. However, this can also be interpreted to mean that physicians in general, regardless of their specialty, tend to wait until a very late stage in the patient’s overall deterioration before making a DNAR decision. When a decision was made without involving the patient, physicians stated that 61% of these patients had not been informed about the decision after it was made while 32% had been informed. The reasons given for not providing information were reasonable but also raise the question of why discussions and decisions about CPR and DNAR were not conducted at an earlier stage. In a Swiss study [28], physicians stated that the reasons for excluding patients was their inability to make decisions, barriers to communication and the concern that such discussions would be emotionally challenging for patients. A positive prognosis (with CPR in the event of cardiac arrest) and the opposite (DNAR) were also given as reasons for excluding patients from the decision-making process [28]. In addition to these barriers, the probable reasons why one third of the patients in the present study were not given information about their DNAR order could be related to discomfort, inadequate training, inexperience and other shortcomings on the part of physicians themselves, as found in a previous review [9].

The proportion of patients who received information regarding DNAR orders appears to vary between countries and clinical settings and is also perceived differently by physicians and nurses. We found that DNAR decisions were discussed with 38% of patients. This contrasts with the study by Bertilsson et al. [26] in which discussions with patients were only conducted in 14% of cases. Varying study populations and methods could partially explain this difference, i.e., there may be a difference between what is documented in the patients’ medical records and the participants’ responses to a questionnaire.

Further, our results show that 41% of physicians stated that DNAR decisions were discussed with patients, compared to 36% of nurses. This lack of consensus about the extent to which decisions were discussed with patients has been confirmed in other studies. 50% of nurses at a Norwegian ICCU reported that they had frequently noted that patients with decision-making ability were not informed about DNAR decisions [15]. Over 55% of Finnish nurses working in neurology, oncology, internal medicine and primary health care reported that DNAR decisions were discussed either “always” or “often” with patients who were able to communicate and understand [7]. Finnish physicians from the same clinical settings stated that DNAR decisions were discussed either “always” or “often”, meaning 72% of cases [29].

There could be several reasons why the nurses in the present study reported significantly lower percentages of patients receiving information. One possible reason is that nurses are sometimes not present when physicians provide information or that, in conversation with the patient or their relatives, the nurse understood that the patient was unaware of the decision and assumed that the physician had not provided any information. A further reason is highlighted by Deep et al. [30] i.e. that the persons present sometimes understand the discussion differently. Differences in interpretation between patients and physicians were found in 20% of all DNAR decisions, resulting in either DNAR decisions being made for patients who did not request them or no DNAR decisions being made for patients who did request them [30].

### Perceptions and attitudes to DNAR orders

In a Swedish study from two decades ago, 53% of the responding physicians and nurses stated that competent patients should be consulted about DNAR decisions [17]. Our findings show that this proportion has increased to 81%. This change in attitude is described in the ERC guidelines as the shift from a doctor-centred focus, emphasizing beneficence, towards one focusing more on the patient’s autonomy [8]. This is also reflected in the Swedish Patient Act in which patient autonomy and the right to participate in decisions regarding their treatment is emphasised, including the right to refuse treatment [22]. Reasons for withholding treatment are further defined in the ethical guidelines of the Swedish National Board of Health and Welfare regarding life-prolonging treatment [23].

81% of participants stated that the patient’s opinion about DNAR should be heard in cases in which the patient is capable of making a decision. This raises the question of why (or when) a capable patient should *not* be heard about a decision that concerns their own care. In a study [1] aimed at assessing attitudes underlying physicians’ decision-making, 535 physicians from Sweden, Germany and Russia participated in a questionnaire presenting three case vignettes about a critically ill 82-year-old Alzheimer’s patient. It demonstrated that Swedish physicians were most likely to withhold life-prolonging treatment, including CPR. For all three countries, the most significant attitude variable affecting a decision was the patient’s level of dementia [1]. In a study aimed at assessing how DNAR decisions are made, communicated and perceived, Gibbs et al. found that factors influencing attitudes towards DNAR decisions included health economics, cultural attitudes towards death and the role of family in society [31].

When asked whether a patient’s desire to receive CPR in the event of a sudden cardiac arrest should always be respected, one third of the professionals answered in the affirmative. This view—always respect the patient wishes—seems to be based on the (mis)interpretation that patient autonomy is the most important of the four principles of medical ethics [32]. This prioritization between the principles might also be regarded as an easy way of resolving a dilemma between a patient’s wishes and the uncertainty regarding the prognosis in individual cases. This autonomy-orientated approach makes it easier to address the principle of do not harm since many professionals might think that the risks of an unsuccessful CPR are negligible compared to not respecting the patient’s wishes, even if they are considered unrealistic. It is likely that in many cases, the pragmatic solution might be a half-hearted and brief attempt at CPR in which a professional might argue that the principles of autonomy and do no harm have been respected. One of the limitations of a questionnaire study as opposed to an interview study is that the ethical reasoning behind the responses cannot be known. It might even be the case that much of the reasoning surrounding ethical principles is actually a combination of feelings and personal views. This limitation is particularly applicable to the question of whether “the decision was ethically right”. Without a more in-depth interview that considers the four principles of medical ethics, the motivation behind the different responses remains unclear.

In Sweden, relatives are not permitted to make DNAR decisions. 70% of participants in the present study agreed with this legal position. However, 19% of nurses still believed that relatives should be permitted to make DNAR decisions. A literature review [32] investigating experiences of discussions about advance CPR decision-making concluded that a trusted person should initiate the discussion. However, the desire for relatives to be involved varied depending on the context, while the timing of the discussion should preferably be earlier in the patient’s illness rather than during an acute phase [33].

## Limitations

The present study has some limitations that must be considered. As DNAR decisions are not confined to cardiology or internal medicine wards, for example, we decided to invite medical practitioners from a wide range of medical specialities to participate in the study. However, such an approach makes it more difficult to make comparisons with the more homogenous populations used in other studies. However, this could also be considered a strength as the results reflect the attitudes, perceptions and practices in a cross-section of Swedish physicians and nurses. It is likely that this also partially explains the low response rate as, based on their workplace, some of the participants may not have had any experience of DNAR decisions. Although this constitutes a selection bias, it could also be argued that those who responded to the questionnaire were more likely to have either relevant knowledge and practical experience or at least an interest in the subject. Another important limitation is that no dropout analysis, except for professions, was performed due to the anonymous nature of the collected data. The results should also be evaluated based on the risk of response bias. Given the topic of our study, there is a risk of social desirability bias, for example, as a result of respondents seeking to present themselves in a favourable light or having expectations regarding the use of the research findings. Finally, there is a potential limitation regarding memory bias. The purpose of asking respondents to recall their latest DNAR discussion was to reduce the risk of memory bias. However, despite this, there is still a risk that their responses could indicate a memory deficiency on the part of the respondent, rather than being an account of the actual practice.

## Conclusions

Swedish healthcare professionals take patient autonomy into account when making DNAR decisions. Nevertheless, as 50% of patients were considered unable to participate in the discussion prior to the decision, questions remain about the timing of patient participation and whether more discussions should have been conducted earlier. Given the uncertainty about timing, the majority of patients deemed competent participated in DNAR discussions. This is a positive trend towards a clearer emphasis on patient autonomy.

## Supplementary Information

**Additional file 1.** Attitudes, perceptions and practices to ‘do-not-attempt-resuscitation’ (DNAR) orders.

## Data Availability

The datasets used and analysed during the current study are available from the corresponding author on reasonable request.

## Abbreviations

CPR: Cardiopulmonary resuscitation
DNAR: Do-not-attempt-resuscitation
