# Supplementary material for: Do-not-attempt-resuscitation orders: attitudes, perceptions and practices of Swedish physicians and nurses
Source: BMC Med Ethics. 2021 Mar 30;22:34. doi: 10.1186/s12910-021-00604-8 (PMC8008584; doi:10.1186/s12910-021-00604-8)
Supplement: Supplementary file 1 — Additional file 1. Attitudes, perceptions and practices to ‘do-not-attempt-resuscitation’ (DNAR) orders. [file 12910_2021_604_MOESM1_ESM.docx]

Attitudes, perceptions and practices to ‘do-not-attempt-resuscitation’ (DNAR) orders

*Please complete the free text fields and tick your responses below. The questionnaire takes around 10 minutes to complete.*

# I. General questions

1. Age _______years

2. Sex ⎕ Male

⎕ Female

⎕ Prefer not to say

3. What is your current professional category/profession?

⎕ Assistant (house) physician before graduation

⎕ Assistant (house) physician before internship

⎕ Medical intern (house officer)

⎕ Licensed physician before specialist residency

⎕ Medical resident

⎕ Medical specialist

⎕ Senior physician

⎕ Head of section/Chief physician

⎕ Registered nurse

⎕ Specialist nurse

⎕ Other professional title

If “Other professional title”, what title?___________________________________________

4. How many years have you been active in your current professional category/profession? _______years

5. How many years have you been active in healthcare in total? _______years

6. Within what kind of speciality/unit do you have your **main** service?

⎕ Medicine (e.g. internal medicine, geriatrics, renal medicine, rheumatology)

⎕ Surgery (e.g. orthopaedics, urology, surgery)

⎕ Infection

⎕ Psychiatry

⎕ Emergency medicine (e.g. ambulance care, emergency ward)

⎕ Intensive care/anaesthesia

⎕ Other speciality/unit

If “Other speciality/unit”, which? _______________________________________________

7a. Are you aware that new ethical guidelines for CPR were published in 2013 by the Swedish Society of Medicine, Swedish Society of Nursing, and the Swedish Resuscitation Council?

⎕ Yes

⎕ No

7b. Have you read these guidelines?

⎕ Yes

⎕ No

⎕ Uncertain

7c. Have the guidelines been presented or discussed at your clinic in another way?

⎕ Yes

⎕ No

⎕ Uncertain

# II. Your experience of DNAR decisions and information on them to the patient

*(If the respondent answers “No” to* ***both*** *questions 8* ***and*** *9, the respondent is referred to* ***question 24.*** *In the online questionnaire, the respondent was automatically directed to question 24).*

8. Have you ever participated in a discussion leading to a DNAR decision?

⎕ Yes

⎕ No

9. Have you ever made a DNAR decision?

⎕ Yes

⎕ No

***Answer questions 10-19 based on the latest discussion about a specific DNAR decision that you participated in:***

10. Was the decision discussed with the patient?

⎕ Yes

⎕ No

⎕ Uncertain

11. Was the prognosis discussed with the patient?

⎕ Yes

⎕ No

⎕ Uncertain

12. Was the patient asked about his or her opinion in the process that led to the DNAR decision?

⎕ Yes

⎕ No

⎕ Uncertain

13. Did the patient have the opportunity/ability to participate in the discussion?

⎕ Yes

⎕ No

⎕ Uncertain

14. Was it the patient him- or herself who initiated the discussion?

⎕ Yes

⎕ No

⎕ Uncertain

15. Was it the patient him- or herself who requested the DNAR order?

⎕ Yes

⎕ No

⎕ Uncertain

16. If the DNAR decision was made without the patient’s participation, was the patient informed of the decision once it was made?

⎕ Yes

⎕ No

⎕ Uncertain

16 b. If you answered “No” to question 16, why was the patient not informed?

*(In the online questionnaire, this question is only displayed if the respondent answered “No”)*

State the reason: _____________________________________________________________

17. Was the decision ethically right?

⎕ Yes

⎕ No

⎕ Uncertain

18. Should the decision have been made earlier?

⎕ Yes

⎕ No

⎕ Uncertain

19. Should the decision have been made later?

⎕ Yes

⎕ No

⎕ Uncertain

# III. Your experience of DNAR decisions and information on them to relatives

***Answer questions 20-23 based on the latest discussion about a specific DNAR decision that you participated in:***

20. Was the decision discussed with the patient’s relatives?

⎕ Yes

⎕ No

⎕ Uncertain

21. Was the patient’s prognosis discussed with the patient’s relatives?

⎕ Yes

⎕ No

⎕ Uncertain

22. Were the patient’s relatives given an opportunity to participate in the discussion?

⎕ Yes

⎕ No

⎕ Uncertain

23. Was it the patient’s relative who initiated the discussion?

⎕ Yes

⎕ No

⎕ Uncertain

# IV. Your opinions of information about an advance decision to refrain from attempting resuscitation in the event of sudden cardiac arrest

24. Should the patient’s opinion about DNAR always be requested on condition that the patient is capable of making a decision?

⎕ Yes

⎕ No

⎕ Uncertain

25. Consider a situation in which the patient has expressed a strong desire to receive CPR in the event of sudden cardiac arrest. Should this desire always be respected, regardless of other medical and ethical assessments?

⎕ Yes

⎕ No

⎕ Uncertain

26. Should previously made decisions regarding DNAR be re-evaluated if the patient’s condition/prognosis improves?

⎕ Yes

⎕ No

⎕ Uncertain

27. Should the opinions of relatives about DNAR always be requested?

⎕ Yes

⎕ No

⎕ Uncertain

28. Should relatives be allowed to make DNAR decisions, regardless of other medical and ethical assessments?

⎕ Yes

⎕ No

⎕ Uncertain

29. Do you think there are patients who **want** to be informed that a DNAR decision has been made by the physician in charge but who **do not** receive such information?

⎕ Yes

⎕ No

⎕ Uncertain

30. Do you think there are patients who **are** informed that a DNAR decision has been made by the physician in charge but who **do not** want such information?

⎕ Yes

⎕ No

⎕ Uncertain

31. What percentage of the patients for whom you have responsibility have a valid DNAR order?

________% (provide an estimation in per cent)

32. What percentage of the patients for whom you have responsibility who have a valid DNAR order are aware of this?

________% (provide an estimation in per cent)

33. What percentage of those who have a DNAR order, **without knowing about it**, do you think could take in this information if it were provided?

_________% (provide an estimation in per cent)

34. Comments:

___________________________________________________________________________

*Thank you for your participation!*
